# The role of macrophage polarization and cellular crosstalk in the pulmonary fibrotic microenvironment: a review

**DOI:** 10.1186/s12964-024-01557-2

**Published:** 2024-03-09

**Authors:** Bo-wen Zhou, Hua-man Liu, Fei Xu, Xin-hua Jia

**Affiliations:** 1 The First School of Clinical Medicine, Shandong University of Traditional Chinese Medicine, Jinan, 250014 China; 2 Department of General Medicine, Affiliated Hospital of Shandong University of Traditional Chinese Medicine, Jinan, 250014 China; 3 Department of Pneumology and Critical Care Medicine, Affiliated Hospital of Shandong University of Traditional Chinese Medicine, Jinan, 250014 China

**Keywords:** PF, Macrophage polarization, Fibroblast, Alveolar epithelial cell, Crosstalk

## Abstract

Pulmonary fibrosis (PF) is a progressive interstitial inflammatory disease with a high mortality rate. Patients with PF commonly experience a chronic dry cough and progressive dyspnoea for years without effective mitigation. The pathogenesis of PF is believed to be associated with dysfunctional macrophage polarization, fibroblast proliferation, and the loss of epithelial cells. Thus, it is of great importance and necessity to explore the interactions among macrophages, fibroblasts, and alveolar epithelial cells in lung fibrosis, as well as in the pro-fibrotic microenvironment. In this review, we discuss the latest studies that have investigated macrophage polarization and activation of non-immune cells in the context of PF pathogenesis and progression. Next, we discuss how profibrotic cellular crosstalk is promoted in the PF microenvironment by multiple cytokines, chemokines, and signalling pathways. And finally, we discuss the potential mechanisms of fibrogenesis development and efficient therapeutic strategies for the disease. Herein, we provide a comprehensive summary of the vital role of macrophage polarization in PF and its profibrotic crosstalk with fibroblasts and alveolar epithelial cells and suggest potential treatment strategies to target their cellular communication in the microenvironment.

## Introduction

Pulmonary fibrosis (PF) is a group of progressive terminal lung diseases that do not currently have an effective treatment. Each year in Europe alone, the number of new cases diagnosed with idiopathic PF (IPF) is more than 40,000 [1], and the average survival of patients with PF is normally 3–5 years after diagnosis [2], although the course of disease varies among individuals. Nonspecific syndromes, such as dry cough and progressive dyspnoea, may cause clinicians to fail to consider the diagnosis of PF in practice [3]. Typically, advanced age (normally > 65 years) is considered a high-risk factor [4] for the development of PF. The aging population globally and increasing rates of hospital admissions also indicate the burden of this disease. Pharmacologic treatments, such as pirfenidone [5] and nintedanib [6], mitigate the worsening of lung function but do not improve the average survival. Therefore, the need for more effective and safe treatments remains a challenge for the management of PF.

Based on the latest research on laboratory animals and clinical experiments, the pathological progress of PF is not only an outcome of chronic inflammatory disorders, but also an unbalanced epithelial damage/injury response following many types of tissue injuries in the lung microenvironment [7]. In parallel, histopathological studies [8] have demonstrated dysregulated macrophage polarization and abnormal proliferation of non-immune cells, such as alveolar epithelial cells (AECs), fibroblasts, and mesenchymal stem cells. Many cellular crosstalk events, which occur intermittently and repeatedly over time, drive uncontrolled senescence, metabolism, and other processes, leading to aberrant wound repair [9]. Following injury to the alveolar epithelium, dysfunction of lung homeostasis, cytokines, and growth factors drive signalling to initiate various repair and immune pathways that contribute to the fibrotic microenvironment, which is characterised by macrophage recruitment, fibroblast accumulation, and extracellular matrix (ECM) deposition. Recent evidence indicates that macrophages exhibit anti-fibrotic properties as a result of the delivery of exosomes to AECs and lung fibroblasts [10] or polarization among different phenotypes [11]. The findings, coupled with experimental data showing that macrophage polarization participates in the lung fibrotic microenvironment and progression through the secretion and submission of pro-fibrotic mediators among nonimmune cells, has increased interest on the role of crosstalk between macrophages and non-immune cells in PF.

In this review, we discuss the latest studies that have shown the unique role of macrophage polarization and the activation of non-immune cells in the pathogenesis and progression of PF, their cellular crosstalk at the molecular level, and various signalling pathways and mechanisms that may be involved in lung fibrosis. Finally, we provide perspective on the remaining questions and future treatment targets within the scope of macrophage polarization and cellular crosstalk in the PF microenvironment.

## Macrophage polarization promotes fibrotic progression

It has been long established that macrophages are the primary messengers in chronic inflammation and fibrotic progression [12], which induce injury and abnormal signals. Several single-cell RNA sequencing studies have investigated the heterogeneity within alveolar macrophages (AMs), fibroblasts, and epithelial cells in the lungs of human donors with PF [13]. These studies have shown that AMs consist of two distinct populations: tissue-resident AMs and monocyte-derived AMs [14]. Single-cell reports have further demonstrated that the selective deletion of pro-fibrotic genes in monocyte-derived AMs accelerates progressive fibrosis. When epithelial injury promotes fibrosis during COVID-19 acute respiratory distress syndrome (ARDS), the accumulation of monocyte-derived macrophages (MDM) has performed a more profibrotic transcriptional gene sets than other macrophage populations [15] against foreign stimulation of the immune system and are activated into different phenotypes. Apart from the classical pro-inflammatory M1 and alternative anti-inflammatory/pro-fibrotic M2 types, many other phenotypes (such as M2a, M2b, M2c, and M2d) participate in cell communication between macrophages and the lung microenvironment by regulating tissue regeneration and growth factor levels [16]. Current investigations of AM behaviour normally fall under the M1–M2 paradigm, which establishes that macrophages can exhibit opposite phenotypes in various disease states by repolarization [16].

### The role of macrophages in PF

Resident tissue macrophages, which are widely recognized for their essential roles in host defense, inflammatory response, and microenvironment homeostasis, consist of AMs in the alveolar space and interstitial macrophages (IMs) in the pulmonary interstitial matrix [17]. Nevertheless, resident tissue macrophages would be replaced by MDMs under the stimulation of inflammation or injury, and the MDMs may take over the function of resident tissue macrophages. As observed in respiratory viral infective models [18], excessive and uncontrolled release of proinflammatory cytokines by monocyte-derived AMs can accelerate lung injury than original AMs. Consequently, the overactivation of macrophages may play a critical role in PF pathogenesis.

As the only cell population exposed to air, AMs are key cells that induce early biological effects in the lungs. By using the CD11b-diphtheria toxin receptor (DTR) in transgenic mice to establish an IL-13-dependent model, Borthwick et al. [19] demonstrated that lung fibrosis and inflammation crucially rely on monocyte-derived AMs to maintain type-2 immunity, and AMs may harness this role by regulating chemokine production and recruiting effector T cells to the lungs. AMs produce various growth factors or cytokines that influence the maintenance of immune responses; however, both biophysical and biochemical signals can also regulate the phenotypes of AMs to facilitate the progression of PF. Usually, the ECM is a structural scaffold for cells to adhere to and receive mechanical stimulation, and its pathological remodelling leads to the transformation of AMs. After seeding with radiation-induced decellularised ECM, AMs polarise to M1 but not M2 via the integrin-dependent activation of nuclear factor kappa B (NF-κB) [20], which aggravates the infiltration of inflammatory cells. In addition to normal inflammatory signals, inflammasomes (e.g., NLRP3) that undergo pyroptosis also contribute to the activation and pro-fibrotic effects of AMs [21, 22]. Furthermore, they accumulate IL-1b and IL-18 in macrophages and thereby stimulate the release of transforming growth factor-beta (TGF-β), which directly triggers fibrosis progression [23].

The pro-fibrotic property of AMs is related to lipid metabolic imbalance, including the ‘cellular response to fatty acids’ and ‘positive regulation of lipid metabolic processes’ [24]. In a murine model, Romero et al. [25] observed that the mRNA levels of lipogenic genes decreased after bleomycin injury but bronchoalveolar lavage (BAL) lipid levels increased. When co-cultured with the BAL lipid extraction, AMs have been observed to polarise into the M2 phenotype and display excessive production of TGF-β. The authors deduced that abnormal lipid surfactants accumulated and were oxidised in the alveolar space and then mediated the transformation of AMs into foam cells, leading to the final pro-fibrotic phenotype. It has also been reported that apolipoprotein E (ApoE) from monocyte-derived AMs impairs the progression of lung fibrosis by inducing Collagen I phagocytosis in vitro and in vivo; this process depends on the key lipid receptor low-density lipoprotein receptor-related protein 1 [26]. These findings suggest that the modulation of the lipid metabolism process through AMs may be a novel target for treating PF.

Compared with AMs, IMs were only discovered a few decades ago, which is partly due to the small number of cells and the inability to collect them from samples such as bronchoalveolar lavage fluid (BALF) [27]. Using single-cell transcriptome technology, various subsets of IMs have been isolated from normal murine lungs [28], and two IMs subpopulations arising from tissue-recruited monocytes express higher levels of genes contributing to wound healing, repair, and fibrosis. Simultaneously, it has been identified that the secreted levels of IL-6 and IL-10, as well as IL-1 receptor antagonist (IL-1Ra) were higher in IMs than in AMs, which suggests that IMs are an anti-inflammatory phenotype in disease [29]. Moreover, IMs isolated from radiation-induced lung fibrosis mouse models exhibit a pro-fibrotic ability by activating myofibroblast differentiation, and the specific depletion of IMs has been shown to inhibit fibrotic progress in vivo [30]. IM isolated from murine models have demonstrated higher expression of arginase (Arg)-1, a marker of the M2 phenotype, than AMs, and IMs with specific depletion with a CSF1R neutralising antibody exerts anti-fibrotic activity. However, it is still unclear whether macrophages have the same characteristics and transformation properties in humans as in mice. The role of these cells after chronic inflammation also remains unclear [31].

### Modulation of macrophage polarization in PF

Considering macrophages are highly heterogeneous plastic cells in the microenvironment, they are believed to play vital roles not only in pulmonary homeostasis but also in inflammatory and fibrotic progression. Macrophages can transform from one phenotype to another, or even vice versa, under cytokine stimulation [32]. According to the classic M1/M2 macrophage paradigm, researchers normally use the term ‘polarization’ to define this perturbation of macrophages with multiple stimuli producing various patterns of gene and protein expression [33]. A growing body of evidence supports the role of M1/M2 macrophage polarization in the pathogenesis of PF.

M1 macrophage polarization mostly activates inflammatory processes which can be classically induced by LPS and IFN-γ. By inducing and releasing pro-inflammatory cytokines with chemokines such as IL-1β and TNF- α, M1 macrophage can mediate tissue injury and initiate an inflammatory response [34]. Myocyte enhancer factor 2C can modulate M1 macrophage polarization in an IL-12-dependent manner [35]. Moreover, using public mouse transcriptomic data, Orecchioni et al. analysed and compared different gene signatures between activated M1 macrophages in vivo and in vitro and demonstrated that M1-polarised mice responded by upregulating many pro-inflammatory genes that are positively correlated with IL12/arginase [36]. Upregulated genes, both in vivo and in vitro, were actively involved in the modulation and polarization of macrophages. Interestingly, their expression is mediated by the Janus kinase (*Jak2*) JAK/signal and signal transducer and activator of transcription (*Stat1/Stat2*) pathway [37]. The findings were further supported by studies indicating that JAK inhibitors (e.g., ruxolitinib, tofacitinib, and itacitinib) can exert anti-inflammatory effects on M1 macrophages and modulate lung fibrogenesis in a model of HOCl-induced ILD by diminishing the expression of polarised markers and pro-inflammatory cytokines, such as CD86, MHCII, IL-6, and TNFα [38]. Thus, the polarization of M1 macrophages in lung injury serves as an indicator of a modest inflammatory response with high levels of pro-inflammatory cytokines associated with Th1/Th2 immune responses [39]. Nevertheless, a sustained inflammatory response would provoke the progression of interstitial fibrosis. In the early stages of inflammation and aberrant healing, there is enrichment of pro-fibrotic exudate macrophages [40] and polarization from AMs to M1 macrophages, as observed in microscopic sections [41]. Another study found that the Mo and/or Cd induced injury may cause macrophages to polarise toward M1 via the TLR4/NF-κB/NLRP3 pathway, leading to lung fibrosis progression by increasing the expression levels of TGF-β1, Smad2, and Smad3 [42]. Collectively, the experimental data verify the specific role of M1 macrophage polarization in the early inflammatory features of PF; however, it is still unclear whether M1 macrophage polarization participates in other phenotypes that promote lung fibrosis. The mechanical contributions of the matrix, oxidative stress, and mitochondrial dysfunction to M1 macrophage polarization require further investigation.

Cumulative evidence has shown that the M2 rather than the M1 phenotype is dominant during the pathological process of PF [43]. From the favoured conceptual model of the lung fibrotic progress, it has been posited that M2 macrophage polarization promotes the secretion of pro-fibrotic mediators, especially TGF-β, which lead to the deposition of interstitial fibrosis [44]. Wang et al. demonstrated a dramatic increase in the number of M2 macrophages in the BALF and lung tissues of mice with fibrosis induced by high tidal volume–mechanical ventilation [45]. The results also showed that persistent tilt polarization toward M2 macrophages was associated with epithelial–mesenchymal transition (EMT) by upregulating the expression of TGF-β1 and p-Smad2/3. In that regard, AMs have been shown to promote SiO_2_-induced PF in a series of chain reactions including those undergoing M2 polarization, synthesising into TGF-β1 precursors, and then transforming into myofibroblasts [46]. However, multiple studies have investigated the modulative effects of cytokines and immune cells on M2 macrophages in the lung microenvironment. The polarization of macrophages toward the M2 phenotype can be induced by Th2 cytokines such as IL-4 and 13 [47]. Moreover, NKT cells have been shown to exhibit a protective role in a bleomycin-induced model by decreasing the Th2 milieu, inhibiting M2 polarization, and eventually alleviating PF [48]. Several independent lines of evidence suggest that the dysregulated action of matrix metalloproteinases (MMPs) implicated in PF may play a central role in its associated pathogenesis. MMP-10 can promote macrophage migration and induce macrophage polarization into an M2 phenotype through the upregulation of collagenase activity [49]. In bleomycin-treated mice, excessive MMP-28 expression polarises M1 macrophages into M2 phenotypes and activates fibroblast proliferation and collagen synthesis, boosting fibrotic progression [50]. Such events ultimately disrupt the balance between pro-fibrotic and anti-fibrotic mediators, resulting in an aberrant repair process in PF [51].

**Fig. 1:**
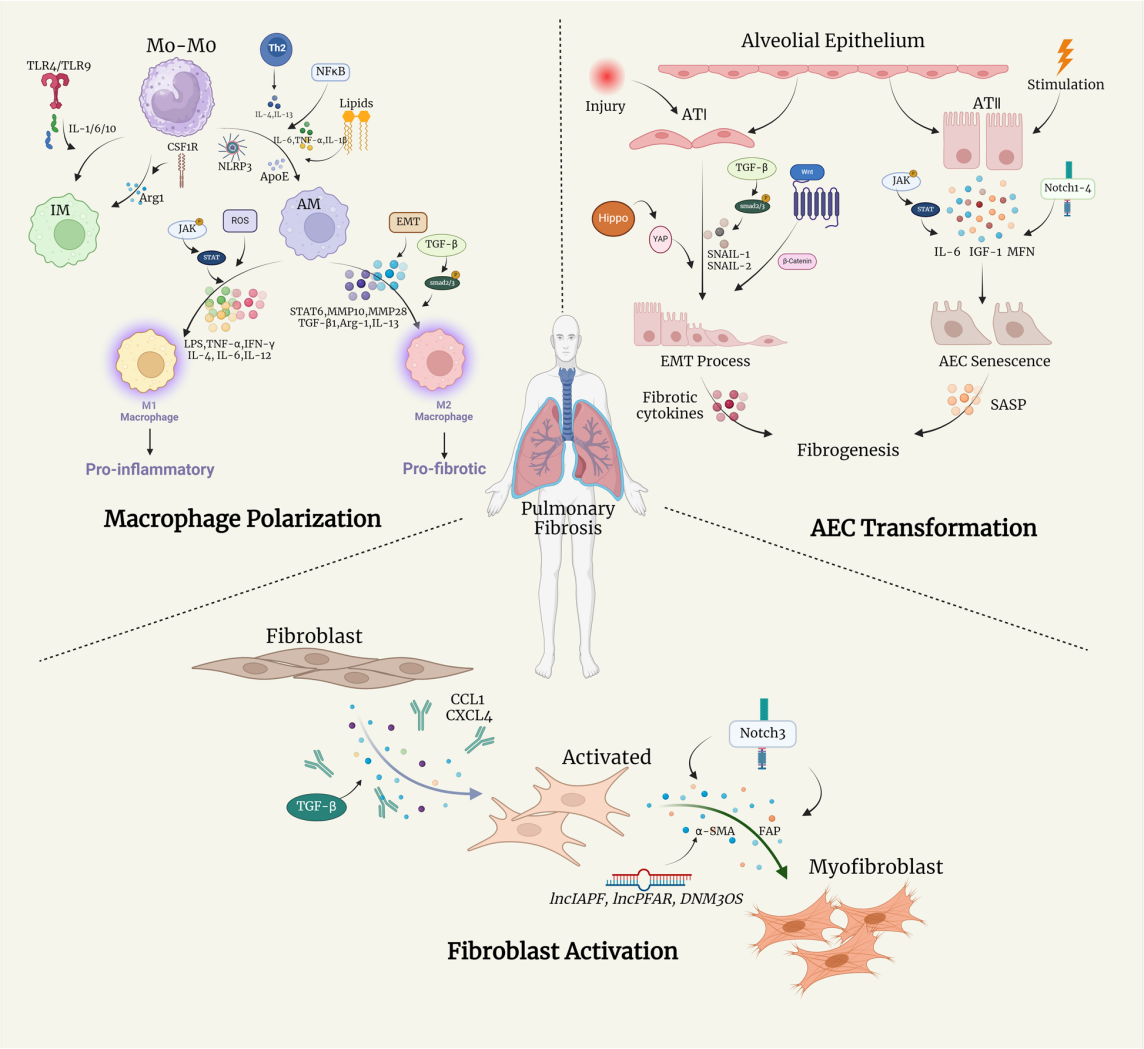
Schematic overview of major mechanisms and key regulators in macrophage polarization, fibroblast activation and AEC transformation within PF microenvironment. (IL: interleukin; ECM: extracellular matrix; TGF-β1: transforming growth factor-beta; TNF α: Tumor Necrosis Factor-α; NF κB: nuclear factor kappa-light-chain-enhancer of activated B-cells; NLRP3: NLR Family Pyrin Domain Containing 3; ApoE: apolipoprotein E; LPR1: lipoprotein receptor-related protein 1; TLR: Toll-like receptors; CSF1R: clinically available colony-stimulating factor receptor-1; LPS: lipopolysaccharides; IFN-γ: interferon-γ; MEF2C: myocyte enhancer factor 2 C; STAT6: Signal Transducer And Activator Of Transcription 6; MMP: matrix metalloproteinases; CXCR4: C-X-C receptor 4; CXCL: C-X-C motif ligand; ECM: extracellular matrix; YAP: Yes-Associated Protein; TAZ: Tafazzin; FMT: fibroblast-to-myofibroblast transformation; PI3K: phosphatidylinositol 3-kinase; FAP: fibroblast activation protein; JNK: JUN N -terminal kinases; ECM: extracellular matrix; JNK: JUN N -terminal kinases; WISP-1: WNT1inducible-signaling pathway protein 1; SASP: senescence-associated secretory phenotype; IGF-1: insulin like growth factor 1; JAK/STAT: Janus tyrosine Kinase-Signal Transducer and Activator of Transcription; MFN: mitochondrial fusion proteins mitofusin)

Furthermore, macrophage polarization is widely believed to be a dynamic process. In this regard, hyperactive cytokines and immune cells in the lung microenvironment contribute to pro-fibrotic polarization of macrophages, which aggravates the imbalance between the M1 and M2 phenotypes, eventually leading to progressive PF. This complementary relationship between various macrophages could be a target for future treatment strategies. Specifically, blocking specific macrophage-polarised pathways may aid in the treatment of PF. All significant mechanisms and regulators mentioned above in macrophage polarization through PF progress are illustrated in Fig. 1 and Table 1.

**Table 1:** The inducers of macrophage polarization in pathogenesis of PF

Macrophage phenotype	inducer	Mechanism of action	Pathway	References
AM	IL-13	Recruit T cell to lung Levels of IL-4/IL-13↓	Type-2 immunity	[19]
	ECM	M1 activation↑ Expression of IL-6/TNF-α/IL-1β↑	NFκB	[20]
	NLRP3	Accumulated IL-1β/IL-18 induce TGF-β↑	NLRP3	[21, 22]
	oxidized phospholipids	Increasing abnormal lipid surfactant promoted M2 polarization↑	Lipids metabolism	[24]
	ApoE	Expression of Collagen I↑	LPR1	[26]
IM	TLR4/TLR9	Increased IL-6/IL-10/IL-1 receptor antagonist↑	Toll-like receptor	[29]
	CSF1R	Higher level of Arg-1 than AMs↑	CSF1/CSF1R	[30]
M1	LPS	Secretion of pro-inflammatory IL-6/ TNF-α↑	LPS/TLR4	[34]
	MEF2C	Expression of IL-12a/IL-2b↑	IL-12	[35]
	LPS/IL-4	Gene levels of IL-6/IL-9/IL-15↑	IL12/Arg	[36]
	IFN-γ	Gene levels of JAK1/JAK2/TYK2↑	JAK/STAT	[37]
	LPS/IFN-γ	Levels of CXCL10, IL-6 and TNFα↑	JAKs	[38]
	ROS	Upregulation TGF-β1, Smad2, Smad3, COL1A1, α-SMA, and MMP2↑ Downregulation of Smad7 and TIMP2↓	TGF-β/Smad2/3	[42]
M2	TGF-β1 and p-Smad2/3	Upregulation of TGF-β1 and p-Smad2/3 promotes EMT↑	TGF-β1/Smad2/3	[45, 46]
	STAT6	Upregulation of IL-4, IL-13 and STAT6↑	IL4/IL-13	[47]
	IL-4, 5, and 13	IL-4, 5, and 13 protein synthesis decrease Arg-1↓	NKT/Th2	[48]
	MMP-10	Promoted M2 phenotypes with increased collagenase activity↑	Collagen degradation	[49]
	MMP-28	Enhanced IL-6↑ Downregulation of Arg-1 and Ym1↓	EMT	[50]

*↑* Increase, *↓* Decrease, *IL* Interleukin, *ECM* Extracellular matrix, *TGF-β1* Transforming growth factor-beta, *TNF-α* Tumor Necrosis Factor-α, *NF-κB* Nuclear factor kappa-light-chain-enhancer of activated B-cells, *NLRP3* NLR Family Pyrin Domain Containing 3, *ApoE* Apolipoprotein E, *LPR1* Lipoprotein receptor-related protein 1, *TLR* Toll-like receptors, *CSF1R* Clinically available colony-stimulating factor receptor-1, *LPS* Lipopolysaccharides, *IFN-γ* Interferon-γ, *MEF2C* Myocyte enhancer factor 2 C, *STAT6* Signal Transducer And Activator Of Transcription 6, *MMP* Matrix metallopro

## The role of cellular crosstalk in the PF microenvironment

### Activation of fibroblasts in PF

Considering chronic epithelial injury and dysfunctional healing both trigger the fibrotic process, fibroblasts, one of the major non-immune cell types involved in wound healing, play a critical role in the PF microenvironment. Based on scRNA-seq analysis and DNA methylation of PF fibroblast phenotypes, activated myofibroblasts are increasingly viewed as heterogeneous, with profibrotic properties [52, 53]. The activation of unremitting fibroblasts in PF consists of fibroblast-to-myofibroblast transformation (FMT), migration, resistance to apoptotic clearance, and excessive deposition of ECM proteins. Further research has highlighted the potential application of myofibroblasts as a primary indicator of ECM deposition in fibrosis and as PF effector cells with a fibroblast-synthesizing capacity [54, 55]. In addition, previous in vitro studies have shown that several chemokines, including CCL12, CXCL4, and CCL1 [56–58], drive the fibrotic process by recruiting and activating fibroblasts at different PF stages. Furthermore, there is a strong link between ECM stiffness and TGF-β1-induced fibroblast differentiation, which suggests the capacity of the ECM to dictate FMT processes and promote progressive PF [59]. Recent reports have also indicated that the close association between long non-coding RNAs (lncRNAs) and PF development contributes to fibroblast activation, particularly by acting as key mediators, similar to lncIAPF [60], lncPFAR [61], and lncRNA DNM3OS [62], which regulate autophagy or cellular crosstalk. The evolving concept with fibroblast activation mechanisms underlying PF is regulated by multiple signalling pathways simultaneously. For example, the Notch signalling cascade has been involved in lung myofibroblast differentiation [63]; particularly, the deficiency of Notch3 has protected lung tissues from fibrotic injury by reducing collagen deposition and α-SMA-positive myofibroblast release following bleomycin administration [64, 65]. In view of the significance of Notch3 in the activation of fibroblasts, it might be a therapeutic target inhibiting Notch3 against PF. Furthermore, the inhibition of phosphatidylinositol 3-kinase (PI3K) on fibroblast activation protein would suppress the production of hydroxyproline (a major building block of collagen), reduce collagen deposition, and increase mice survival [66]. The reactivation of the Wnt/β-catenin pathway is also associated with pro-fibrotic cellular functions, including EMT and myofibroblast differentiation in the PF microenvironment [67]. Therefore, stimulation of the above signalling molecules or pathways may impact the initiation of the transformation of fibroblasts into myofibroblasts in the lung interstitium, and delineation of the communication between macrophages and fibroblasts throughout PF would lead to better and more specific medication-based treatment strategies. All significant mechanisms and regulators mentioned above in the activation of fibroblasts through PF progress are illustrated in Fig. 1 and Table 2.

**Table 2:** The inducers of fibroblast activation and alveolar epithelial cells transformation in the pathogenesis of PF

Type	Inducer	Mechanism of action	Pathway	References
Fibroblast activation	TGF-β1	TGF-β1 induced the upregulation of DNMT3a and TET3 expression in fibroblasts↑	DNA methylation	[55]
		Inhibited PDGFRα or PDGFRβ blunt macrophage infiltration and differentiation into myofibroblasts↓	PDGFR	[56]
	CXCL4	Boosting myofibroblast differentiation and collagen synthesis↑	CXCL4	[57]
	CCL1	Recruiting fibroblasts via CCR8↑ Mediating fibroblast activation via AMFR	AMFR-SPRY1	[58]
	ECM	Overexpression of YAP, TAZ and glycoprotein fibronectin dictates FMT process↑	FMT	[59]
	*lncIAPF*	Inhibited ELAVL1/HuR dependent autophagy to regulate FMT↓	ELAVL1/HuR	[60]
	*lncPFAR*	Acting as a competing endogenous RNA of miR-138 to promote fibroblasts activation↑	YAP1-Twist	[61]
	*DNM3OS*	Acting as a fibroblast-specific critical downstream effector of TGF-β-induced lung myofibroblast activation↓	miRNA	[62]
	Notch3 FAP	Notch3 deficiency mitigates PF via inhibiting myofibroblast activation↓	Notch3	[64]
		α-SMA-positive myofibroblasts increased after Notch3 knockout↓	Notch3	[65]
Fibroblast activation		FAP targeted PI3K inhibitor against myofibroblasts activation and collagen deposition↓	PI3K	[66]
	Wnt/β-catenin	Enhanced EMT and myofibroblast differentiation↑	Wnt/β-catenin	[67]
Alveolar epithelial cells	TGF-β	Elevated TGF-β promotes the EMT inducers and maintainer, SNAIL-1 and SNAIL-2↑	TGF-β/SMAD	[68]
	WISP-1	Upregulation of WISP-1 activates ECM-related cytokines to induce fibrotic process↑	Wnt/β-catenin	[69]
	YAP	Transmitting the mechanical tension to fibrotic phenotypes within PF microenvironment↑	Hippo	[70]
	Notch1-4	Notch activity regulates EMT interactions crucial for development and homeostasis of primary AECs↑	Notch	[71]
	SASP	Remodel profibrotic microenvironment↑ Enhancing ECM components during fibrosis↑	AEC senescence	[72]
	IL-6	Elevated the expression of p16 and p21 in aged ATII cells↑	JAK/STAT	[73, 74]
	IGF-1	Mediated the expression of p21 andβ-Galactosidase in aged ATII cells after radiation↓	AEC senescence	[75]

*↑* Increase, *↓* Decrease, *DNMT3a* DNA methyltransferase 3 alpha, *TET3* Tet methylcytosine dioxygenase 3, *PDGFR* Platelet-derived growth factor (PDGF)-receptor, *CXCR4* C-X-C receptor 4, *CXCL* C-X-C motif ligand, *ECM* Extracellular matrix, *YAP* Yes-Associated Protein, *TAZ* Tafazzin; FMT: Fibroblast-to-myofibroblast transformation, *IL* Interleukin, *PI3K* Phosphatidylinositol 3-kinase, *FAP* Fibroblast activation protein, *JNK* JUN N -terminal kinases, *ECM* Extracellular matrix, *JNK* JUN N -terminal kinases, *TGF-β* Transforming growth factor-beta, *WISP-1* WNT1inducible-signaling pathway protein 1, *SASP* Senescence-associated secretory phenotype, *IGF-1* Insulin like growth factor 1, *JAK/STAT* Janus tyrosine Kinase-Signal Transducer and Activator of Transcription, *MFN* Mitochondrial fusion proteins mitofusin

### Macrophage-fibroblast crosstalk in PF

Several metabolites and soluble paracrine factors produced by macrophages are conventionally viewed as essential mediators in the biological switch between macrophage polarization and fibroblast activation, or macrophage-fibroblast crosstalk, in the pathological progression of PF. S100a4, also known as fibroblast-specific protein-1, was initially regarded as a protein specifically expressed by fibroblasts, whereas current experiments indicate that it can induce mesenchymal progenitor cell fibrogenicity in idiopathic PF [76] and also coincides with the presence of macrophages [77]. S100a4 expression was significantly up-regulated by M2 polarised AMs from IFN-γR^−/−^ mice with MHV-68. Conditioned media with the presence of recombinant S100a4 protein increase the production of pro-fibrotic cytokines such as α-SMA and collagen I in primary mouse lung fibroblasts after 24 and 48 h of exposure, which ultimately promotes the pathogenesis of lung fibrosis via fibroblast activation, migration, and FMT [78]. Overall, the results suggest that S100a4 released from macrophages contributes to PF by promoting fibroblast differentiation. Nevertheless, the abundant expression of CX3CR1 in macrophages has prompted researchers to evaluate the role of the CX3CL1–CX3CR1 axis in PF [79]. Although CX3CR1 deficiency may not have an impact on the macrophage population after BLM administration, it can still affect macrophage polarization, and subsequent fibrotic progresses, such as myofibroblast activation. By utilising co-culture models to analyse the interaction between macrophages and fibroblasts, Li et al. have explained the mechanisms underlying macrophage-induced PF that macrophage-derived TSLP and MMP9 jointly contribute to lung fibrosis by promoting EMT process and fibroblasts migration [80].

In some conditions, neither pro-inflammatory cytokine deficiency nor steroid and immunosuppressive therapies can limit PF. Based on the current research, it is vital to consider the possibility that the presence of an immunoregulatory microenvironment, which mainly comprises regulatory lymphocytes and myeloid cells, may be associated with lung pathological processes. Within the type-2 immune response, IL-10 is an inducer of fibrogenic particles, especially M2-like and Th2-like pro-fibrotic cells [81]. Consistent with the major profibrogenic cytokine TGF-β1, it has been found that the anti-inflammatory cytokine IL-10 is produced by alveolar macrophages, and another study further demonstrated that IL-10 suppresses lung fibrosis in a TGF-β1-dependent manner, wherein the overexpression of IL-10 simultaneously regulates the M2 polarization via the CCL2/CCR2 axis [82]. In this regard, it has been suggested that immunoregulatory cytokines, typically TGF-β1 and IL-10, are major participants in the pro-fibrotic microenvironment, which trigger a series of fibroproliferative wound healing processes and are relevant to inappropriate communication between M2-like polarised macrophages and activated fibroblasts in lung tissues [83]. In both the BLM-induced rat and fluorescein isothiocyanate-induced mouse models, chronic exposure to microcystin-leucine arginine (LR) contributed to an amelioration of PF [84]. Further in vivo data indicated that microcystin-LR, as an immune regulator, can attenuate macrophage polarization toward the CD206 + M2-like phenotype, thereby blocking UPR^ER^ signal transduction in stressed cells, which may be a mechanism underlying the inhibition of pulmonary EMT and FMT. The difference between macrophage subpopulations in normal and fibrotic lungs supports the idea of a specific role of innate immune factors in the pathogenesis of lung fibrosis. To emphasise this point, co-localisation and causal modelling have been utilised to verify macrophages that highly express *SPP1* and *MERTK* (SPP1^hi^), and these identified macrophages are crucial in accelerating the activation of myofibroblasts in lung tissues and in the upregulation of stress response-associated genes [85]. Emerging evidence indicates that ER stress induces various pro-fibrotic functions through inducing interactions between myofibroblasts and macrophage polarization. Germline mutations have induced ER stress and activated a signalling network known as the unfolded protein response (UPR), which could also be regulated by PI3K and C/EBP homologous protein (CHOP) [86, 87].

Considering intercellular communication through metabolites has been previously defined in cancer cells and carcinoma-associated fibroblasts [88], it is necessary to provide insight into the role of immunomodulators in PF as an intercellular message between macrophages and fibroblasts. Glycolytic escalation in lung fibroblasts, smooth muscle cells, and endothelial cells are responsible for the pathological progression of lung fibrosis [89]. Subsequent analysis has indicated that there is a significant increase in glycolytic-related lactate in the conditioned media of TGF-β1-induced lung myofibroblasts and mice BALFs [90]. Macrophages treated with myofibroblast-conditioned media have switched to pro-fibrotic phenotypes with a high level of selected pro-fibrotic mediators which establish myofibroblast glycolysis and accelerate the pro-fibrotic activity of macrophages in lung fibrosis. Fatty acid (FA) metabolism in cells regulates various biological activities [91] and energy production through FA oxidation. Altered contents and profiles of FA metabolism have been identified in patients with PF and in animal models, suggesting its critical role in the development of pro-fibrotic phenotypes in macrophages and fibroblasts/myofibroblasts [92]. For example, the polarization of macrophages to the M2 phenotype is not only dependent upon the energy provided by FA [93] but also activated by the transcription factor peroxisome proliferator-activated receptor (PPAR)-γ, which was initially recognised in adipose tissue for its role in FA storage [94]. Additionally, cell lineage tracing to lung fibroblasts in mice has shown that both large quantities of pro-fibrotic cytokines from polarised macrophages and PPAR-γ can induce phenotype switching between lipofibroblasts and myofibroblasts during the progression and resolution phases of lung fibrosis [95]. Exosomes and their enriched microRNAs (miRNAs) have collectively merged as potent modulators in complex networks that communicate between neighbouring or distant cells [96], and their utility as biomarkers of PF has been explored [97]. To investigate whether macrophage-derived exosomes are able to transfer anti-fibrotic miR-142-3p to target cells, Guiot et al. co-cultured MRC5 cells with purified THP1 macrophage-derived exosomes for 24 h and treated the cells with TGF-β for 4 h [10]. They found that the delivery of miR-142-3p from macrophage-derived exosomes to fibroblasts can reduce TGFβ-R1 transcription the expression of and pro-fibrotic genes, which may eventually slow the progression of PF. Although there are still several challenges to overcome before pharmacological application [98], exosomal miRNAs have been found to be potential biomarkers for fibrogenesis, diagnosis, and therapeutic intervention in lung fibrosis [99].

### Transformation of alveolar epithelial cells in PF

In the alveolar lumen, alveolar epithelial cell type I (ATI), alveolar epithelial cell type II (ATII), and interalveolar septa mix in the continuous epithelium lining in the alveolar surface. Histopathological evidence has shown that aberrant epithelial cells, especially hyperplasia, and a lack of ATI cells, are some of the defining features of IPF, suggesting that the variability of ATII cells, which develop into ATI phenotypes, might add a heavy burden to fibrotic lung tissues due to mesenchymal expansion [100, 101]. In addition, there is consensus that ATII cells maintain alveolar niche homeostasis by the secretion of surfactant and to act as progenitor cells for regeneration or differentiation into ATI phenotypes in PF pathogenesis [102]. Recent reports have further demonstrated that EMT-associated signalling and senescence pathways regulate ATII cell transformation during fibrotic airway injury and remodelling. EMT in ATII cells is a progressive process, along with the deficiency of E-cadherin and other cytokines, as well as the alteration of cell migration, adhesion, and expression of ECM components, which are sustained by specific signalling pathways, such as the TGF-β/SMAD2/3 signalling loop [68] and Wnt/β-catenin pathways [69]. Furthermore, Hippo and Notch signalling in dysregulated ATII cells promote both ATII plasticity for transdifferentiation into a pro-fibrotic phenotype and epithelial diminished renewal by transmitting mechanical tension from alveolar epithelium to myofibroblasts [70, 71, 103]. Cell senescence, which is observed in lung fibrosis, has been demonstrated to play a fundamental role in the pathogenic alterations of ATII cells [104]. The fibrotic capacity of senescent ATII cells can mediate signals to mesenchymal cells within the same PF microenvironment via the acquisition of a specific senescence-associated secretory phenotype (SASP) or epithelium-derived triple type 2 cytokines [72, 105]. In addition, upregulation of the IL-6 pathway by JAK/STAT signalling has been shown to drive senescent gene transcription both in IPF epithelial cells and fibroblasts [73, 74]. Meanwhile, the loss of IGF-1 receptor in radiation-induced model has inhibited the capacity of senescent ATII cells to polarise macrophages into the M2 phenotype via IL-13, one of the central SASPs in the PF microenvironment [75]. Furthermore, several cell-autonomous factors are involved in the exhaustion of ATII cell self-renewal and their capacity to recruit inflammatory cells, especially AMs [106]. The above evidence largely suggests that alveolar epithelial cells have a direct role in the fibrotic microenvironment and cellular crosstalk. All significant mechanisms and regulators mentioned above in the transformation of alveolar epithelial cells through PF progress are illustrated in Fig. 1 and Table 2.

### Crosstalk between macrophages and the alveolar epithelium in PF

As the alveolar epithelium lies at the interface between the external environment and internal haemostasis, repeated exposure to injury and stimulation requires accurate modulation of inflammatory reactions in response to pro-fibrotic cellular crosstalk. In the PF microenvironment, polarised macrophages participate in a complex crosstalk network with alveolar epithelial cells, particularly ATII cells, via several mechanisms and cytokines. The addition of IL-4 and IL-13 to bone marrow-derived macrophage media has been found to be an M2-polarising reagent [107]. Thus, Hult et al. chose a combination of IL-4 and IL-13 to acquire M2 culture media and found that the expression of fibrotic factors (platelet-derived growth factor alpha, connective tissue growth factor) and antiapoptotic mediators (COX and BCL2) were upregulated in ATII cells treated with M2 culture media [108]. However, the differences in apoptosis between AEC cultured in M1 or M2 media are limited, suggesting that AEC apoptosis relies on juxtacrine signalling from macrophages in the pro-fibrotic microenvironment. Furthermore, by comparing macrophage populations from murine strains with different fibrosis-prone properties, Chung et al. observed that superoxide production from the M2 phenotype partially depends on NOX2 in fibrotic progenesis, and the significant increase in these superoxides promotes M2 macrophages to induce alveolar epithelial cell senescence, which ultimately increases the susceptibility of a patient to radiation-induced lung fibrosis [109]. Importantly, the infiltration of macrophages might be the first step of the early PF stage due to their aggregation occurring earlier than the EMT process. Li et al. analysed macrophage-secreted cytokine levels after bleomycin treatment, and the data indicated that the inhibited PI3K/Akt signalling was involved in the macrophage-induced EMT progress between alveolar epithelial cells and fibroblasts within the PF microenvironment [110]. Spontaneously, the key genetic variant in IPF pathogenesis could also alleviate the fibrotic crosstalk between macrophages and AECs, in which keratin 8 knockdown in murine IPF models downregulated the expression of macrophage chemokines and further inhibited macrophage-induced transitional AEC senescence from bleomycin exposure [111]. Based on a co-culture system consisting of macrophages, epithelial cells, and fibroblasts, Ni et al. demonstrated that the knockout of STING signalling in macrophages impairs M1 polarization and inhibits the fibrotic response in fibroblasts, which emphasises the significance of STING signalling in the cellular crosstalk between macrophages, lung epithelial cells, and fibroblasts [112].

As described above, AECs have been found to have a direct role in pro-fibrotic macrophage activation; however, the mechanisms underlying macrophage polarization and recruitment that depend on epithelial signalling or cytokines in the PF microenvironment have not yet been fully elucidated. Epithelial cells have been implicated in the migration of pathological macrophages. In a recent in vivo study, bleomycin-induced AEC insult led to a significant increase in CCL2 and CCL12; in such cases, monocyte-oriented macrophages were recruited by these chemokines in the lung and ultimately promoted the exudate phenotype associated with lung fibrosis [113]. Evidence supporting this cellular crosstalk comes from an early fibrotic in vivo study, wherein the migration of bleomycin-treated Ly6C monocytes was promoted to inflamed tissues by CCL2 secreted from epithelial cells and subsequently differentiated into increased interstitial macrophages [114]. Additionally, for the purpose of exploring the significant capacity of GM-CSF in fibrogenic AM compartments and surfactants [115], Gschwand et al. analysed the expression of its receptor Csf2 in perinatal and adult mouse lungs. Subsequent data demonstrated that timed expression of GM-CSF in ATII cells switched on the differentiation of foetal AMs, and the absence of ATII-derived Csf2 led to AM population atrophy [116].

**Fig. 2:**
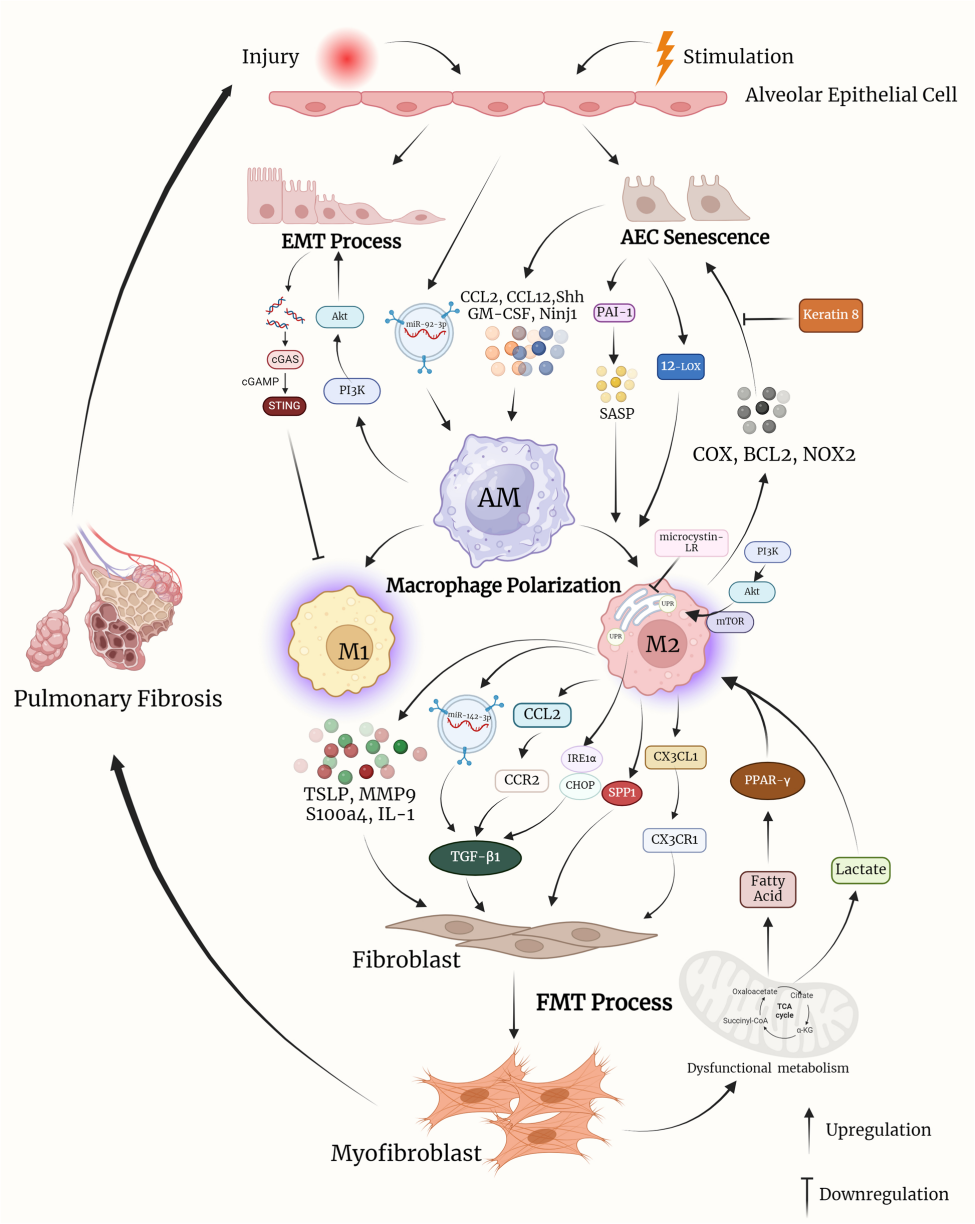
Schematic overview of major mechanisms and key regulators in cellular crosstalk within PF microenvironment. (FMT: fibroblast-to-myofibroblast transformation; ECM: extracellular matrix; TSLP: thymic stromal lymphopoietin; MMP9: matrix metalloproteinase 9; IL: interleukin; LR: leucine arginine; UPR: endoplasmic reticulum unfolded protein response; EMT: epithelial-mesenchymal transition; IRE1α: requiring enzyme 1α; CHOP: C/EBP homologous protein; PPAR-γ: peroxisome proliferator-activated receptor-γ; AEC: alveolar epithelial cell; IL: interleukin; ATII: alveolar epithelial cell type II; COX: Cyclooxygenase; BCL2: B-cell lymphoma-2; PI3K/Akt: Phosphatidylinositide 3-kinases/ protein kinase B; STING: stimulator of interferon genes; GM-CSF: Granulocyte–macrophage colony-stimulating factor; PAI-1: plasminogen activator inhibitor-1; SASP: senescence-associated secretory phenotype; Ninj1: nerve injury-induced protein 1; Shh: Sonic hedgehog; 12-LOX: 12-lipoxygenase)

Alveolar epithelial cells are essential inducers and modulators of macrophage fibrotic activation and polarization. To determine whether senescent ATII cells influence AMs, Rana et al. analysed the alteration in secreted cytokines due to TGF-β1 induced ATII cell senescence and the key role of plasminogen activator inhibitor-1 (PAI-1). Deletion of PAI-1 in senescent ATII cells not only inhibited the stimulatory capacity of ATII-oriented SASP on AM gene expression, but also attenuated the expression of fibrotic mediators (such as IL-4, IL-13, and PDGF) from TGF-β1 induced ATII cell senescence [117]. Liu et al. identified critical effects of AEC-derived exosomes on AM activation; specifically, LPS-induced exosomes from impaired AECs could be efficiently absorbed by AMs to induce the expressions of the inflammatory cytokines, including TNF-α, IL-6, and IL-1β, compared to control groups [118]. Choi et al. discovered increased transmembrane nerve injury-induced protein 1 (Ninj1) expression in bleomycin-induced AECs; the macrophage bond to the AECs was subsequently activated, indicating that Ninj1 may promote the activation of macrophages in a fibrotic murine model by boosting their interaction with impaired AECs [119]. According to bleomycin-induced in vivo and in vitro models, fibrosis-associated macrophage phenotypes are locally similar to those of ATII cells, contributing to paracrine-induced macrophage polarization. For example, sonic hedgehog secreted by ATII cells promotes the alternative activation of osteopontin-mediated macrophages in an autocrine or paracrine manner, thus justifying the epithelial cell-dependent mechanisms underlying M2 polarization and fibrotic pathogenesis [120]. To evaluate the potential role of 12-lipoxygenase (12-LOX) in radiation-induced murine fibrosis, Chung et al. compared the different levels of M2 phenotype biomarkers from primary macrophages cultured in ATII media between wild-type and 12-LOX-deficient mice and found that senescent ATII cells may facilitate macrophage polarization into the preferential M2 type via the 12-LOX dependent pathway in the PF microenvironment [121]. Hence, the crosstalk loop between macrophages and epithelial cells in lung tissues may be involved in the pathogenesis of PF by mediating cytokine, chemokine, and pro-fibrotic pathways within the PF microenvironment. All significant mechanisms and regulators mentioned above in cellular crosstalk through PF progress can be consulted in Fig. 2 and Table 3.

**Table 3:** The mechanism of macrophage cellular crosstalk in the pathogenesis of PF

Type of crosstalk	Mediator	Mechanism of action		References
Macrophage to fibroblast	S100a4	M2-derived S100a4 promoted mesenchymal progenitor cell fibrogenicity and following fibroblast activation, migration, as well as FMT↑		[76–78]
	CX3CR1	The downregulation of M2 polarization induced by CX3CL1-CX3CR1 interaction modulate fibrocytes migration and subsequent myofibroblasts activation↓		[79]
	TSLP/MMP9	Macrophage-derived TSLP/MMP9 promoted the EMT and FMT progress between epithelial cells and fibroblasts↑		[80]
	IL-1/ TGF-β1	Macrophage-secreted IL-10 regulated M2 polarization via CCL2/CCR2 axis and following fibroblast activation in a TGF-β1 dependent manner↑		[81–83]
	microcystin-LR	Chronic exposure of microcystin-LR suppressed the M2 differentiation by blocking UPR^ER^ signaling, thereby inhibiting EMT and FMT↓		[84]
	*SPP1*/*MERTK*	Highly proliferation of *SPP1*^*hi*^ macrophage contributed to the activation of myofibroblast in PF microenvironment↑		[85]
	ER stress/UPR	The enhancement of ER stress upregulated UPR-associated proteins (IRE1α/ CHOP) to promote M2 polarization and facilitated the TGF-β–mediated myofibroblasts differentiation via PI3K/AKT/mTOR pathway↑ ER stress modified macrophage-fibroblast crosstalk by TLR4 and PINK↑		[86, 87]
	miR-142-3p	The delivery of miR-142-3p from macrophage-derived exosomes to fibroblasts can reduce TGFβ-R1 transcript and profibrotic genes expression		[10]
Fibroblast to macrophage	Lactate	Myofibroblast glycolysis relies on lactate to mediate the pathogenic phenotype of alveolar macrophages and induce profibrotic mediator expression in macrophages↑		[89, 90]
	Fatty acid	Dysfunctional fatty acid metabolism in fibroblasts promoted the secretion of PPAR-γ to activate M2 polarization and subsequent phenotypes switch between lipofibroblasts and myofibroblasts↑		[92–94]
Macrophage to AEC	IL-4/IL-13		IL-4/IL-13 induced M2 cell culture media promoted the expression of fibrotic factors and antiapoptotic meditators (COX, BCL2) in ATII cells↑	[108]
	NOX2		M2 macrophage stimulated the senescence of AECs in a NOX2 dependent manner via increasing superoxide production↑	[109]
	PI3K/Akt		Macrophage-secreted cytokine levels are involved in the activation of EMT process through PI3K/Akt signaling↑	[110]
	Keratin 8		Keratin 8 prohibited macrophage-induced transitional AEC senescence from bleomycin exposure by decreasing macrophage fibrotic cytokines↓	[111]
	STING		The blocking of STING signaling inhibited M1 polarization and fibrotic response within the co-culture system of macrophages, fibroblasts and AECs↓	[112]
AEC to macrophage	CCL2/CCL12		AEC-induced CCL2/CCL12 expression recruited monocyte-oriented macrophages within fibrotic microenvironment↑ Senescent AECs promoted Ly6C monocytes to migrate and differentiate into IMs via CCL2↑	[113, 114]
AEC to macrophage	GM-CSF		Expression of GM-CSF in ATII cells would switch on the differentiation of fetal AMs, and the absence of ATII-derived Csf2 led to AM population atrophy↑	[116]
	PAI-1		The deletion of PAI-1 in senescent ATII cells inhibited the secretion of SASP and subsequent M2 polarization↑	[117]
	miR-92a-3p		miR-92a-3p in AEC-derived exosomes could be absorbed by AMs to induce inflammatory cytokines↑	[118]
	Ninj1		Upregulation of Ninj1 in AECs boosted the activation of macrophage within PF microenvironment↑	[119]
	Shh		Shh secreted by ATII cells promoted the alternative activation of osteopontin-mediated M2 macrophage↑	[120]
	12-LOX		Senescent ATII cells may facilitate macrophage to polarize into preferential M2 type via 12-LOX dependent pathway in PF microenvironment↑	[121]

*↑* Increase, *↓* Decrease, *FMT* Fibroblast-to-myofibroblast transformation, *ECM* Extracellular matrix, *TSLP* Thymic stromal lymphopoietin, *MMP9* Matrix metalloproteinase 9, *IL* Interleukin, *LR* Leucine arginine, *UPR* Endoplasmic reticulum unfolded protein response, *EMT* Epithelial-mesenchymal transition, *IRE1α* Requiring enzyme 1α, *CHOP* C/EBP homologous protein, *PPAR-γ* Peroxisome proliferator-activated receptor-γ, *AEC* Alveolar epithelial cell, *IL* Interleukin, *ATII* Alveolar epithelial cell type II, *COX* Cyclooxygenase, *BCL2* B-cell lymphoma-2, *PI3K/Akt* Phosphatidylinositide 3-kinases/ protein kinase B, *STING* Stimulator of interferon genes, *GM-CSF* Granulocyte–macrophage colony-stimulating factor, *PAI-1* Plasminogen activator inhibitor-1, *SASP* Senescence-associated secretory phenotype, *Ninj1* Nerve injury-induced protein 1, *Shh* Sonic hedgehog, *12-LOX* 12-lipoxygenase

## Conclusions and outlook

PF is a progressive and irreversible disease with an unknown cause that severely threatens the prognosis and quality of life of patients. Although small-molecule drugs such as pirfenidone or nintedanib are used widely in clinics, current Western medicine treatments only alleviate the decline of the patient’s lung function and cannot reverse the entire pathological process. The pathogenesis of lung fibrosis involves the abnormal repair of lung tissues, differentiation and proliferation of fibroblasts, and activation and inflammatory responses of immune cells, especially macrophages [122]. A review of the research has revealed a feedback loop between macrophage polarization and non-immune cell activities, including those of fibroblasts and AECs, which regulate pro-inflammatory and pro-fibrotic activities in PF. In the early stage of lung fibrosis, the polarised M1 phenotype may be dominant owing to its direct role in modulating the abnormal immune response to repetitive wound healing by regulating pro-inflammatory cytokines. Emerging evidence has demonstrated that the macrophage polarization into M2 phenotype can be induced by multiple cytokines, chemokines, inflammasomes, and even autophagy in the pulmonary fibrotic microenvironment; therefore, the M2 polarization ultimately promotes the expression of several mediators of epithelial injury and FMT, such as TGF-β1. Fibroblast proliferation can promote macrophage polarization via metabolic reprogramming or ER stress. AECs play two roles in the development of lung fibrosis development; first, their impaired self-renewal, abnormal senescence, and recruitment of inflammatory mediators directly contribute to fibrogenesis mechanisms; and second, their cellular crosstalk with local primary macrophages promotes Mo-M0 migration and M2 phenotype polarization after injury-induced stimuli, which are indirectly involved in the maintenance of the PF microenvironment. Thus, within the lung fibrosis microenvironment, pro-fibrotic macrophage polarization and its primary or subsequent dysfunctional crosstalk with AECs and fibroblasts contribute to the imbalance of immune homeostasis throughout the whole process of PF, which suggests a novel target for an anti-fibrotic approach.

Here, we summarise and highlight the complex dual role of macrophage polarization in the pathogenesis of lung fibrosis, emphasising the therapeutic possibility of regulating cellular crosstalk between macrophages, AECs, and fibroblasts. However, several studies are still needed to develop treatments for patients experiencing PF-associated symptoms. Due to multiple setbacks in pure anti-inflammatory PF clinical trials, the investigation of macrophage regulation has been reoriented and we present a potential approach involving integration of macrophage polarization and cellular communication. Recent reports have similarly suggested that modulating cell–cell circuits may reset macrophage pools within inflammatory episodes [123]. Therefore, it may be worth exploring the strategies that directly inhibit or reprogram such fatal cellular communications rather than sample antibody-mediated therapies. Moreover, future studies should aim to clarify the phenotypic and functional heterogeneity underlying the crosstalk loop between macrophages and other immune cells via genetic, genomic, and metabolomic tools. Such studies could unravel several pharmacological targets for the treatment of PF with impressive safety and efficacy.

## Data Availability

No datasets were generated or analysed during the current study.

## Abbreviations

PF: Pulmonary fibrosis
IPF: Idiopathic PF
AEC: Alveolar epithelial cell
ECM: Extracellular matrix
AM: Alveolar macrophages
IM: Interstitial macrophage
DTR: Diphtheria toxin receptor
NF-κB: Nuclear factor kappa B
TGF-β: Transforming growth factor-beta
BAL: Bronchoalveolar lavage
BALF: BAL fluid
ApoE: Apolipoprotein E
IL-1Ra: IL-1 receptor antagonist
EMT: Epithelial–mesenchymal transition
MMP: Matrix metalloproteinase
FMT: Fibroblast-to-myofibroblast transformation
lncRNA: Long noncoding RNA
FAP: Fibroblast activation protein
PI3K: Phosphatidylinositol 3-kinase
JNK: C-Jun N-terminal kinase
ATI/II: Alveolar epithelial cell type I/II
SASP: Senescence-associated secretory phenotype
TSLP: Thymic stromal lymphopoietin
ER: Endoplasmic reticulum
PINK: PTEN-induced putative kinase
LR: Leucine arginine
UPR: Unfolded protein response
IRE1α: Enzyme 1α
CHOP: C/EBP homologous protein
FA: Fatty acid
PPAR-γ: Peroxisome proliferator-activated receptor-γ
miRNA: MicroRNA
PAI-1: Plasminogen activator inhibitor-1
12-LOX: 12-Lipoxygenase
